# Remote monitoring of cardiorespiratory signals from a hovering unmanned aerial vehicle

**DOI:** 10.1186/s12938-017-0395-y

**Published:** 2017-08-08

**Authors:** Ali Al-Naji, Asanka G. Perera, Javaan Chahl

**Affiliations:** 1 School of Engineering, University of South Australia, Mawson Lakes, SA 5095 Australia; 2 Electrical Engineering Technical College, Middle Technical University, Baghdad, Iraq; 3 Joint and Operations Analysis Division, Defence Science and Technology Group, Melbourne, VIC 3207 Australia

**Keywords:** Unmanned aerial vehicle, Imaging photoplethysmography, Canonical correlation analysis, Video magnification technique

## Abstract

**Background:**

Remote physiological measurement might be very useful for biomedical diagnostics and monitoring. This study presents an efficient method for remotely measuring heart rate and respiratory rate from video captured by a hovering unmanned aerial vehicle (UVA). The proposed method estimates heart rate and respiratory rate based on the acquired signals obtained from video-photoplethysmography that are synchronous with cardiorespiratory activity.

**Methods:**

Since the PPG signal is highly affected by the noise variations (illumination variations, subject’s motions and camera movement), we have used advanced signal processing techniques, including complete ensemble empirical mode decomposition with adaptive noise (CEEMDAN) and canonical correlation analysis (CCA) to remove noise under these assumptions.

**Results:**

To evaluate the performance and effectiveness of the proposed method, a set of experiments were performed on 15 healthy volunteers in a front-facing position involving motion resulting from both the subject and the UAV under different scenarios and different lighting conditions.

**Conclusion:**

The experimental results demonstrated that the proposed system with and without the magnification process achieves robust and accurate readings and have significant correlations compared to a standard pulse oximeter and Piezo respiratory belt. Also, the squared correlation coefficient, root mean square error, and mean error rate yielded by the proposed method with and without the magnification process were significantly better than the state-of-the-art methodologies, including independent component analysis (ICA) and principal component analysis (PCA).

## Background

Unmanned aerial vehicles (UAVs) or drones, particularly small UAVs capable of hover are a rapidly maturing technology with increasing numbers of innovative applications. The ability of a UAV to detect and measure the vital signs of humans can have many applications, including: triage of disaster victims, detection of security threats and deepening the context of human to machine interactions.

Remote-sensing imaging systems provide a convenient way to monitor human vital signs without any physical restrictions. Imaging Photoplethysmography (iPPG) is one of the most promising methods that uses a video camera as a photodetector to detect optical properties passing through or reflecting from the skin due to cardiac synchronous variations. The traditional contact monitoring methods, such as ECG, pulse oximeter, and respiratory belt transducer, require patients to wear adhesive sensors, electrodes and chest straps, potentially for a long time which may be discomfort, infection or adverse reactions in patients with sensitive skin (e.g., neonates or those suffering burns) [1–6]. The desire to solve the problems associated with contact monitoring systems has led to research using video cameras as a non-contact sensor for monitoring of vital signs.

Non-contact methods based on iPPG provide a low-cost and comfortable way to measure vital signs. For example, Takano and Ohta [7] used a time-lapse image acquired from a CCD camera to extract cardiorespiratory signals of stationary subjects under different illumination levels. They used image processing techniques, including auto-regressive (AR) spectral analysis combined with a 1st-order derivative and a 2 Hz low pass filter to analyse changes in the image brightness of the region of interest (ROI) around the cheek in the facial area which allowed detection of both heart and respiratory rates. Later, Verkruysse et al. [8] could remotely extract PPG signals from the RGB channels obtained from a digital camera under ambient light conditions. They used a fast Fourier transform (FFT) and band-pass digital filtering on the PPG signal extracted from the human face to detect heart and respiratory rates. Similarly, in [9, 10] Poh et al. reported on the development of a non-contact and automated method for measuring of the cardiac pulse from the human face recorded using a built-in webcam. They applied a blind source separation (BSS) method based on independent component analysis (ICA) on the RGB channels intensity to obtain three components and used Fourier transformer and band-pass filtering on these components to extract the signal of interest. According to their outcomes, the green component was the best component to extract the cardiac pulse signal. Later, a study by Lewandowska et al. [11] proposed a contactless method for heart pulse monitoring with a webcam based on principle component analysis (PCA) to reduce computational complexity compared to ICA used by [9, 10]. Similarly to Poh’s methodology, Kwon et al. [12] used the front-facing camera of a smartphone to extract cardiac pulse signal based on the frequency analysis of the PPG signal. As claimed in previous studies, the main challenges using iPPG method were illumination variations (caused by the lighting conditions of indoor or outdoor environments, intrinsic camera noise and changes in the skin tone) and subject’s movement (of the entire head, but also facial expressions, eye blinking and speech) during the measurements. Research has been performed to solve these limitations. For instance, to remove the challenges of illumination variations, some investigations [13–16] used head motion generated from the blood cycle from the heart to the head via the carotid arteries to extract the cardiac pulse signal based on a ICA [13], PCA [14, 15] and a frame subtraction method [16]. However, the subjects’ motion remained the main challenge in their results. Another example regarding only to improve subject’s motion, Haan and Jeanne [17] extracted the cardiac pulse signals directly from RGB face image sequences captured from a digital camera using a chrominance based iPPG method for a subject exercising on a stationary exercise bicycle and a stepping machine. According to their outcomes, the proposed method was better than ICA used in [9, 10] and PCA used in [11] for both stationary and moving subjects. Another study by Li et al. [18] proposed a novel heart rate measurement method to reduce the noise in the cardiac pulse signal from the recording of face video caused by both illumination variations and subjects’ motions. They used a Normalized Least Mean Squares (NLMS) filter [19] to deal with noise caused by illumination variations and both the Discriminative Response Map Fitting (DRMF) [20] and the Kanade-Lucas-Tomasi (KLT) algorithms [21] to reduce the noise caused by subjects’ motion. Although their method has shown promising results for heart rate under realistic human-computer interaction situations, it led to a higher computational complexity than other methods. Feng et al. [22] used an optical iPPG signal model to remove noise caused by motion artefacts from the PPG signal based on the optical properties of human skin. They proposed an adaptive colour difference method between the red and green channels acquired from a digital camera and used an adaptive bandpass filter (ABF) based on the spectral characteristics of the PPG signal to extract cardiac pulse signal and reduce motion artefacts in the facial ROI. However, more advanced signal processing techniques are needed to improve their results because a colour difference method and ABF may be inefficient when the noise signal falls within the frequency band of interest. Also, the performance of the optical analysis method may be affected by periodic illumination variations. Recently, Chen et al. [23] used a reflectance decomposition method on the green channel and ensemble empirical mode decomposition (EEMD) to separate the real cardiac pulse signal from the environmental illumination noise in the PPG signal from digital camera video of a human face. Their proposed approach outperformed the current state of the art methods [9, 10]. However, subject respiration can affect the decomposition of facial reflectance and thus distort the signal of interest. A study by Cheng et al. [24] demonstrated the feasibility of removing the illumination variation noise from the cardiac pulse signal in the facial ROI (green channel) from webcam videos using a joint blind source separation (JBSS) and EEMD. The main limitations in their study were that all subjects were asked to keep stationary and both facial ROI and background ROI were assumed to have the same illumination variations. In addition, most of the previous studies had considered only the motion artefacts resulting from the subject movement and not those resulting from camera movement. Therefore, to remove the effects of illumination variations, subject’s movement and camera movement, we proposed a combination of a complete ensemble EMD with adaptive noise (CEEMDAN) and a canonical correlation analysis (CCA) to remove noise acquired from these effects in the PPG signal and thus present a robust non-contact method to remotely extract cardiorespiratory signals (heart rate and respiratory rate) using video sequences captured by a hovering UAV.

## Methods

### Experimental setup and data acquisition

**Fig. 1:**
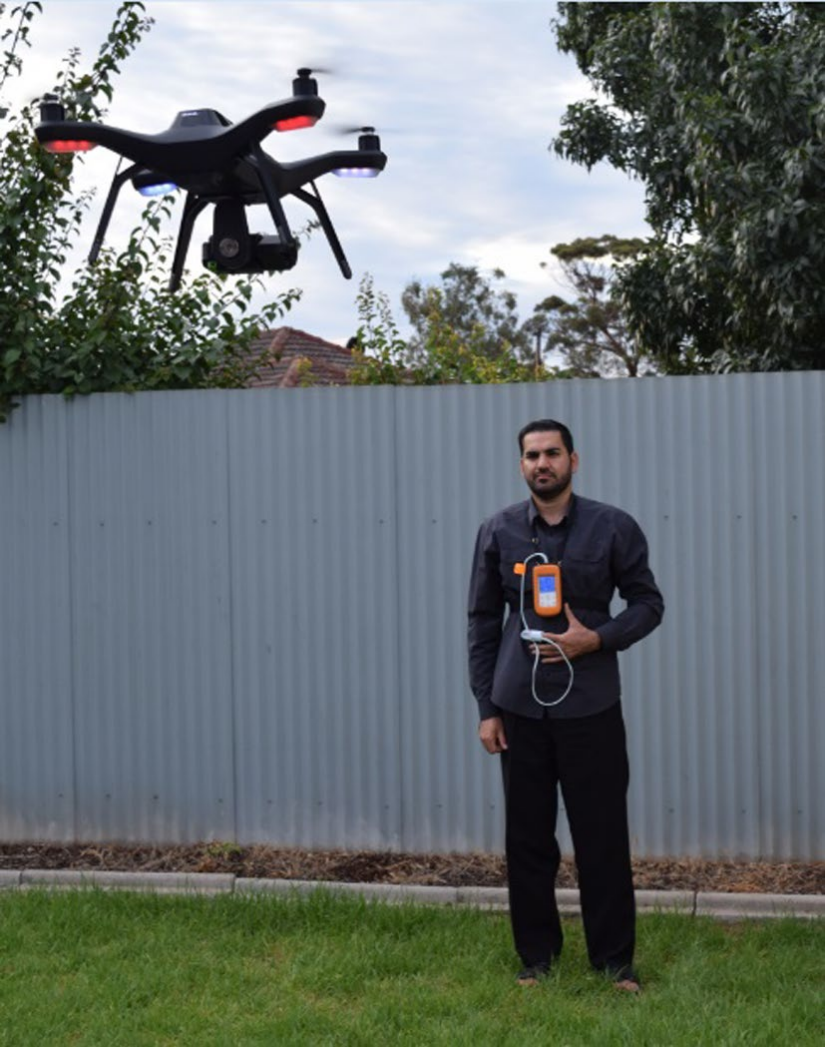
Experimental setup and data acquisition

Fifteen healthy participants (10 males–5 females) with ages ranging from 2 to 40 years were enrolled in the experiment. The ethical approval was granted by the UniSA Human Research Ethics Committee and it was carried out following the rules of the Declaration of Helsinki of 1975. A written informed consent was obtained from each participant before commencing in the experiment. The experiment was performed in the outdoor and indoor environments, where each subject is standing at a distance of 3 m from the front of the UAV camera as shown in Fig. 1. Several videos were acquired for each subject using a hovering UAV (3DR solo) with a GoPro Hero 4k camera at different times of day with different illumination levels. We used a replacement lens (10MP, 5.4 mm GoPro Lens) instead of the original camera lens in order to reduce a fish-eye distortion. Each video was captured at 60 frames per second with a resolution of 1920 × 1080. The video acquisition time was set to 1 min. However, only the last 30 s was chosen for analysis in Matlab program (R2015b). Control measurement of the reference heart and respiratory rates was performed using a finger pulse oximeter (Rossmax SA210) [25] and Piezo respiratory belt transducer (MLT1132) [26] for validation purpose.

### Pre-processing and data analysis

**Fig. 2:**
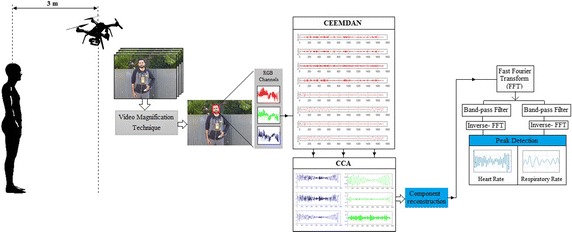
System overview of the proposed

The system framework is composed of five steps as shown in Fig. 2.

**Fig. 3:**
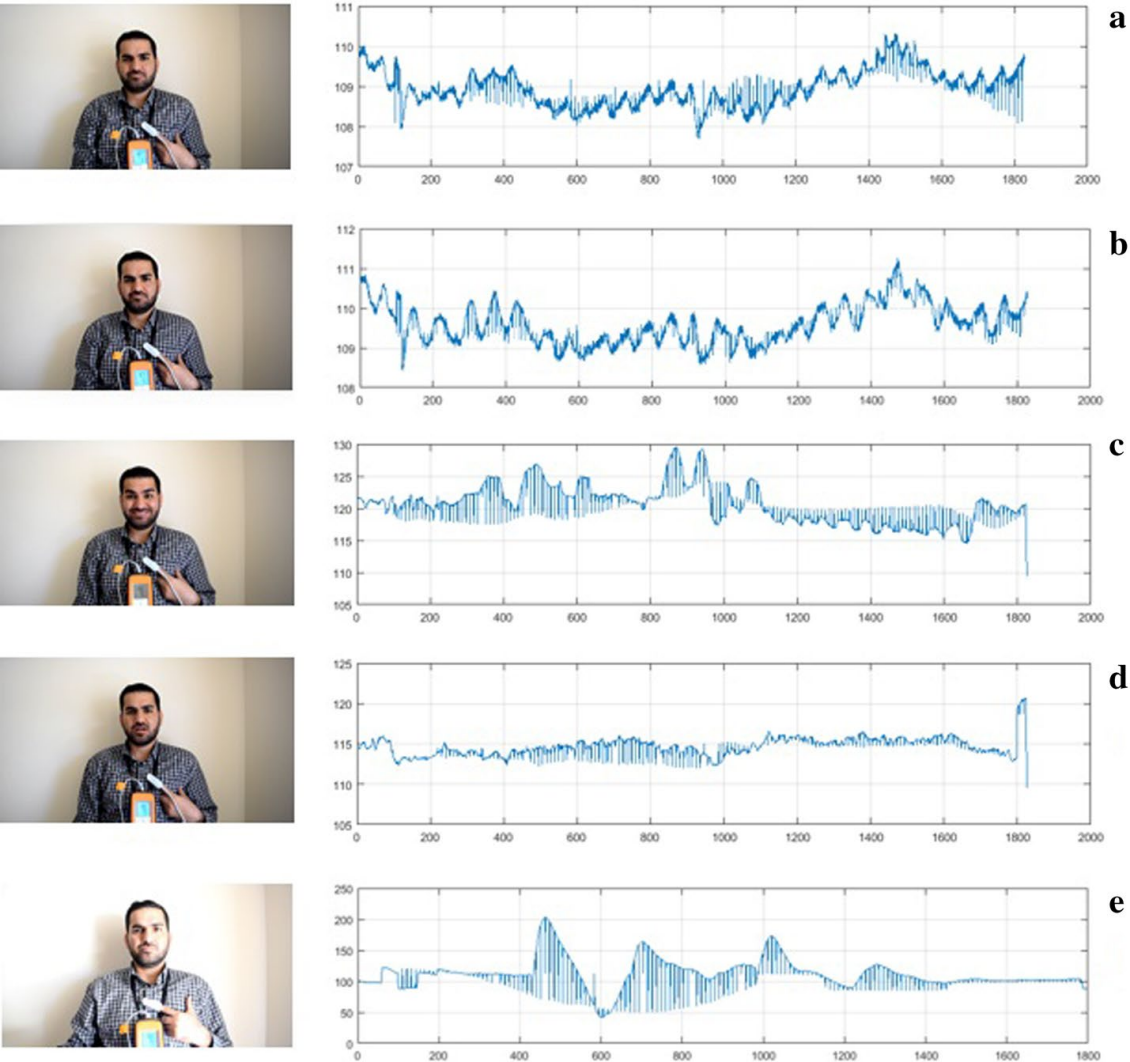
The iPPG signals for facial ROI (green channel) for a subject in case of **a** stationary **b** stationary with 15× magnification, **c** different face expressions, **d** talking, and **e** different illumination conditions

In the first step, we used an enhanced video magnification technique [27] to magnify skin colour variation since the variation caused by the cardiac pulse signal is very weak. Although the digital camera can reveal the iPPG signal, there was substantial noise associated with this signal caused by effecting of illumination variations, subject’s movement and camera movement. We also evaluated the results to examine whether the proposed system with and without magnification process is more efficient than the conventional measurement methods and to improve an importance this process to enhance the iPPG signal. Some examples for iPPG signals acquired from different conditions are given in Fig. 3.

In the second step, to select facial ROI and deal with the problems associated with head movement, we used an enhanced face detection method proposed by Chen et al. [28] instead of Viola–Jones method [29] used in the most previous measurement methods because it was more effective with inclined or angled. Also, the Chen et al. method has a better performance than conventional face detection methods [18, 22, 28]. The raw iPPG signal was then obtained by averaging all the image pixel values within the facial ROI of the green channel as follows:

1 $$iPPG(t) = \frac{{\sum\nolimits_{x,y \in ROI} I \left( {x,y,t} \right)}}{{\left| {ROI} \right|}}$$

where *I*(*x*, *y*) is the pixel value at image location (*x*, *y*), at a time (*t*) and |*ROI*| is the size of the facial ROI.

**Fig. 4:**
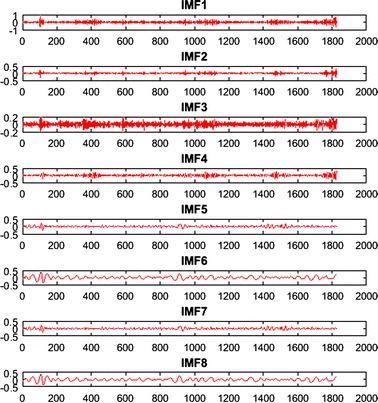
An example of CEEMDAN decomposition of the iPPG signal in the facial ROI

In the third step, we used a complete ensemble EMD with adaptive noise (CEEMDAN) [30], to reduce noise interferences caused by illumination variations from the iPPG signal. CEEMDAN is an advanced signal processing method proposed by Colominas et al. [30] to improve performance of EMD [31] and EEMD [32] by reducing noise from the intrinsic mode functions (IMFs) with more physical meaning. Similar to EMD, CEEMDAN decomposes the original signal into IMFs with instantaneous amplitude and frequency data. An example of eight IMFs decomposition with number of iterations of 200 for ${iPPG (t)}$ is provided in Fig. 4.

**Fig. 5:**
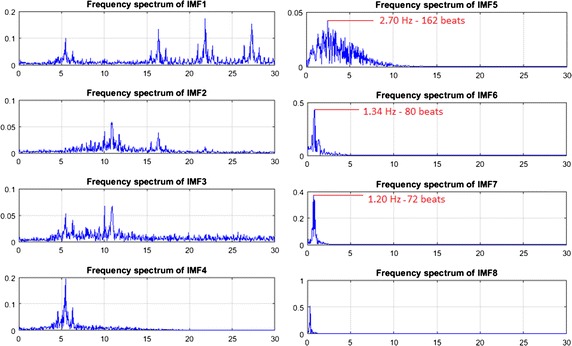
The frequency spectrum of decomposed IMFs

Three IMFs (IMF5, IMF6 and IMF7) were be chosen for estimating cardiorespiratory signals based on their frequency spectra that correspond to the best range of cardiac pulse frequency band as shown in Fig. 5.

Figure 5 shows the spectrum of all IMFs and which IMF has the best frequency bands of interest. It is clear that the frequency bands of IMF5, IMF6 and IMF7 fall within 0.2–4 Hz, corresponding to 12–240 beats/min, whereas the frequency bands of other IMFs fall outside this range. Therefore, just IMF5, IMF6 and IMF7 have been selected as inputs for the next step because they have maximum frequency spectra of 2.7, 1.34 and 1.2 Hz which correspond to 162, 80 and 72 beats respectively.

In the fourth step, CCA technique is then applied on the selected IMFs to remove the motion artefacts components from the iPPG signal. The CCA technique can be used as a blind source separation (BSS) for separating a number of mixed signals [33–35]. This technique is based on second-order statistics to generate components derived from their uncorrelated signals rather than independence components used in ICA. CCA can achieve better performance for BBS than ICA and it has less computational complexity than ICA [36–38].

To understand how CCA works as a BSS method, *j* and *k* are two multi-dimensional random signals. Consider the linear combinations of these signals, known as the canonical variates as follows [33]:

2 $$j = W_{j}^{T} \left[ {j - \overline{\text{j}} } \right],\quad k = W_{k}^{T} \left[ {k - \overline{\text{k}} } \right]$$

where *W*_*j*_ and *W*_*k*_ are weighting matrices of *j* and *k*. The correlation, *ρ*, between these linear combinations is given by

3 $$\rho = \frac{{W_{j}^{T} C_{jk} W_{k} }}{{\sqrt {W_{j}^{T} C_{jj} W_{j} W_{k}^{T} C_{kk} W_{k} } }}$$

where ${{C}}_{jj}$ and ${{C}}_{kk}$ are the nonsingular within-set covariance matrices and ${{C}}_{jk}$ is the between-sets covariance matrix. The largest canonical variates can be found with the maximum value of *ρ* with respect to *W*_*j*_ and *W*_*k*_.

The original green channel signal (*a*) is converted into a multichannel signal (*A*) using the CEEMDAN algorithm. The IMFs determined to be outside frequency bands of interest are removed, and then the remaining IMFs determined to be within frequency bands of interest are used as inputs with the un-mixing matrix *W* of the CCA algorithm. The original multichannel signal $\widetilde{A}$ is then reconstructed without unwanted IMFs (artefact components) using the inverse of the un-mixing matrix *W* ^−1^. Now, the target single-channel signal $\widetilde{a}$ without the noises resulting from the effects of illumination variations, subject’s movement and camera movement can be determined by adding the new IMFs components in the $\widetilde{A}$ matrix.

In the next step, a fast Fourier transformer (FFT) is applied to transform the $\widetilde{{a}}$ signal from the time domain to the frequency domain. Two ideal band pass filters are then used on this signal with selected frequencies of 0.5–4 and 0.2–0.5 Hz corresponding to 30–240 beats/min and 12–30 breaths/min respectively. The inverse FFT is then applied to the result of filtering to obtain the cardiorespiratory signals. Finally, the heart and respiratory rates are measured by using the peak detection algorithm [39].

## Experiments

The experimental results obtained from 15 subjects were set in four scenarios. The first scenario is a stationary scenario, where the subject was standing in front of the UAV without any movement. The second scenario is when the subject was asked to do display different facial expressions during the imagery task with some head rotation. In the third scenario the subject was asked to remain stationary and talk normally during the imagery capture. These three scenarios were set up in outdoor and indoor environments under ambient light conditions. The last scenario is when the imagery sessions were in the indoor environment under different illumination levels. The motion artefacts resulting from a flying UAV camera were included in all proposed scenarios. The frame sequences obtained from the UAV camera for all scenarios were processed through the proposed system with and without the magnification process. We evaluated the performance of the proposed system for heart and respiratory rate measurements with and without the magnification process and compared them with the measurements obtained from ICA [9, 10] and PCA [11] in four scenarios. Also, the statistical analysis based on Bland–Altman method [40] was used to quantify the degree of agreement between these systems and the reference methods (Rossmax Pulse oximeter and Piezo respiratory belt). The mean bias and standard deviation (SD) of the differences, 95% limits of agreement (±1.96 SD), the squared correlation coefficients (CC^2^), root mean squared error (RMSE) and mean error rate (ME) were calculated for the estimated heart and respiratory rates from the proposed systems and the reference methods for all proposed scenarios.

### Heart rate measurements

**Fig. 6:**
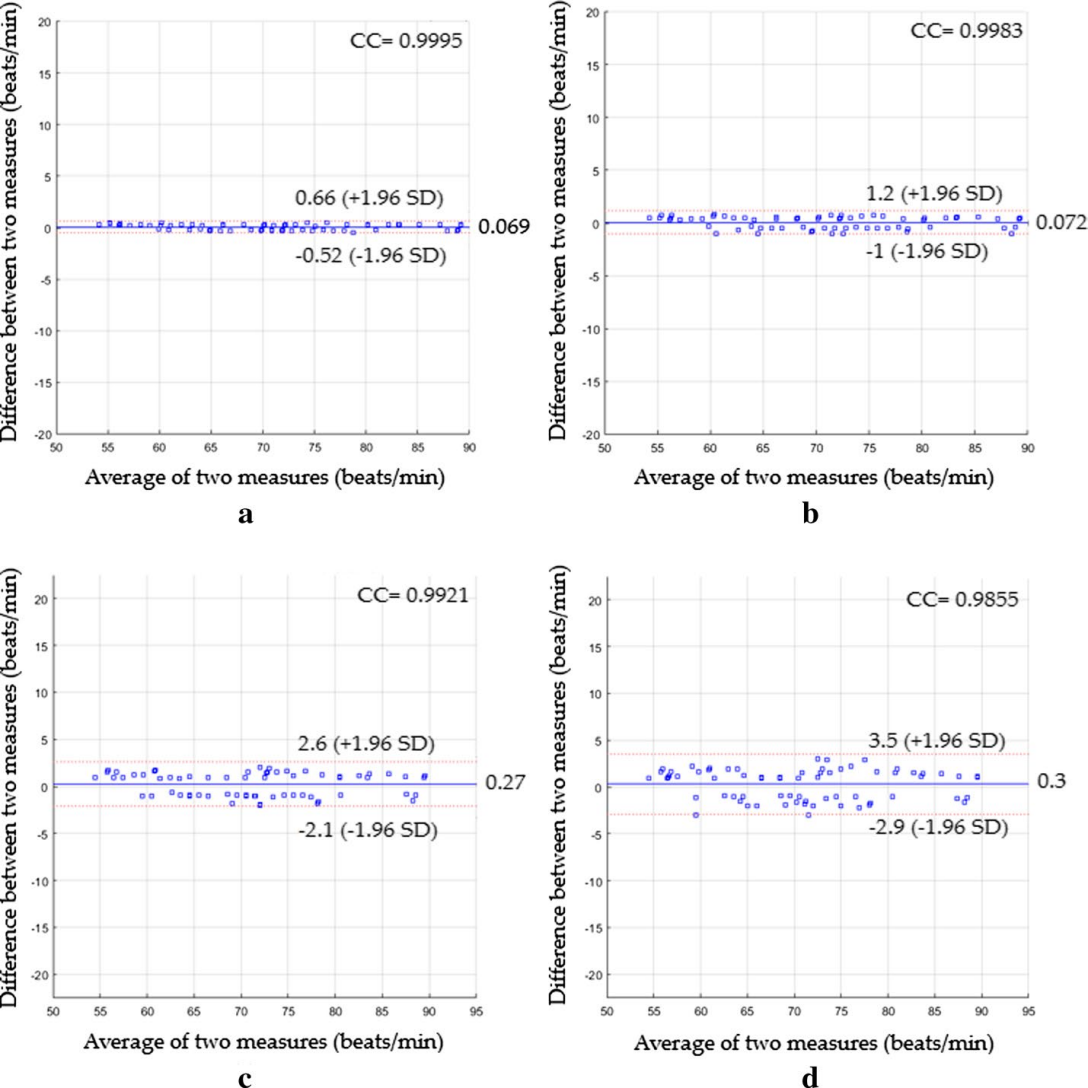
Bland–Altman plots between heart rate measurements obtained by the reference method and heart rates measured by **a** the proposed system with magnification, **b** the proposed system without magnification, **c** ICA and **d** PCA for the first scenario

In the first scenario, the statistical agreement based on Bland–Altman plots of all measuring systems against the reference method (Rossmax Pulse oximeter) is shown in Fig. 6, where the x-axis indicates the mean of the measurements and y-axis is the difference between the measurements.

The Bland–Altman plot based on the proposed system with the magnification process (see Fig. 6a) showed a mean bias of 0.069 beats/min with a lower limit of −0.52 beats/min and an upper 95% limit of +0.66 beats/min with a CC^2^ of 0.9991 and a RMSE of 0.31 beats/min, whereas the Bland–Altman plot based on the proposed system without the magnification process (see Fig. 6b) led a mean bias of 0.072 beats/min with a lower limit of −1 beats/min and an upper 95% limit of +1.2 beats/min with a CC^2^ of 0.9966 and a RMSE of 0.57 beats/min. When the agreement between the heart rate measurements based on ICA was evaluated (Fig. 6c), a mean bias was 0.27 beats/min with 95% limits of agreement −2.1 to 2.6 beats/min, and CC^2^ was 0.9843 with a RMSE of 1.22 beats/min, whereas the statistics were 0.3 beats/min of a mean bias with −2.9 to 3.5 beats/min of 95% limits of agreement, CC^2^ of 0.9712, RMSE of 1.64 beats/min (Fig. 6d) when PCA was used instead.

**Fig. 7:**
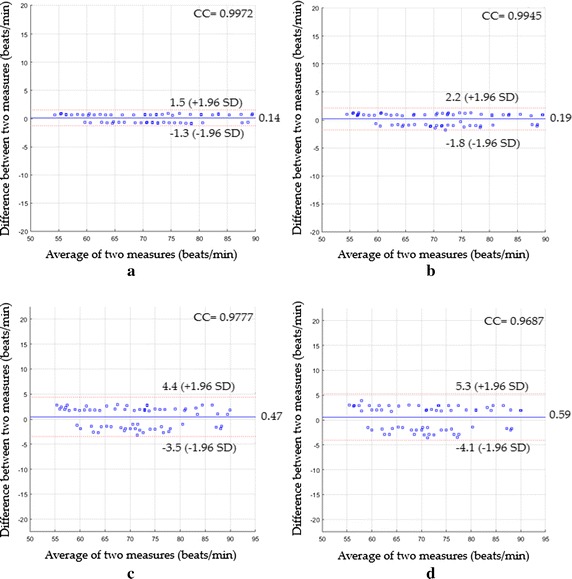
Bland-Altman plots between heart rate measurements obtained by the reference method and heart rates measured by **a** the proposed system with magnification, **b** the proposed system without magnification, **c** ICA and **d** PCA for the second scenario

The Bland–Altman plots for the second scenario are shown in Fig. 7.

As shown in Fig. 7a, a mean bias was 0.14 beats/min and 95% limits of agreement were −1.3 and +1.5 beats/min with a CC^2^ of 0.9945 and a RMSE of 0.73 beats/min. Figure 7b showed that a mean bias was 0.19 beats/min and 95% limits of agreement were −1.8 and +2.2 beats/min with a CC^2^ of 0.9891 and a RMSE of 1.02 beats/min. Using ICA (see Fig. 7c), a mean bias was 0.47 beats/min with 95% limits of agreement −3.5 to 4.4 beats/min and CC^2^ was 0.9559 and RMSE was 2.05 beats/min, while when PCA was used instead, the statistics were 0.59 beats/min of a mean bias with −4.1 to 5.3 beats/min of 95% limits of agreement, CC^2^ of 0.9383, a RMSE of 2.44 beats/min (see Fig. 7d).

**Fig. 8:**
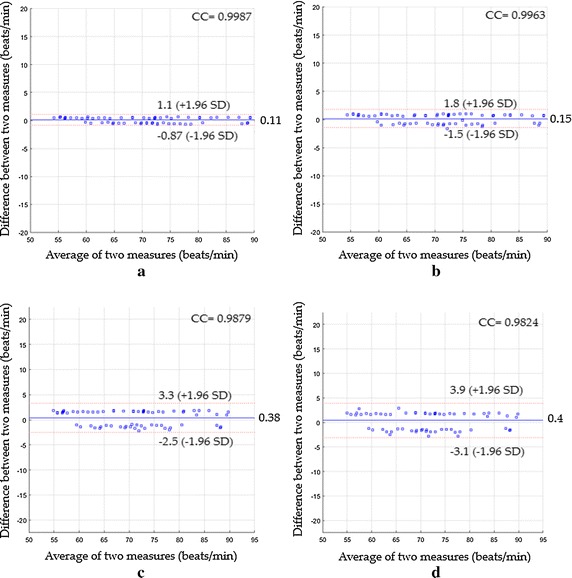
Bland-Altman plots between heart rate measurements obtained by the reference method and heart rates measured by **a** the proposed system with magnification, **b** the proposed system without magnification, **c** ICA and **d** PCA for the third scenario

The Bland–Altman plots for the third scenario are shown in Fig. 8.

**Fig. 9:**
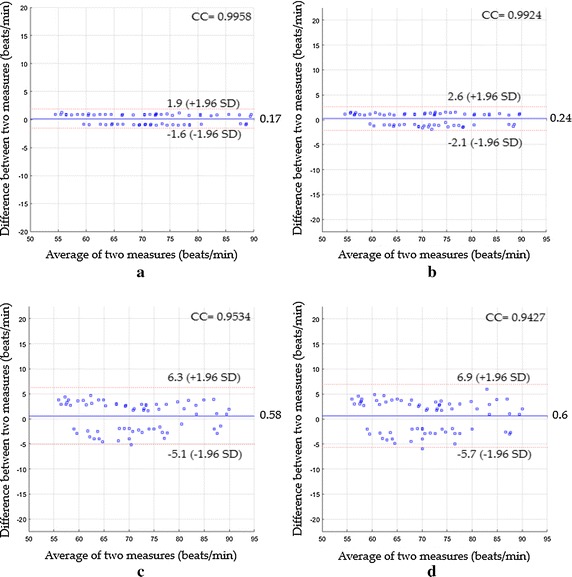
Bland-Altman plots between heart rate measurements obtained by the reference method and heart rates measured by **a **the proposed system with magnification, **b** the proposed system without magnification, **c** ICA and **d** PCA for the fourth scenario

Figure 8a revealed a mean bias of 0.11 beats/min with 95% limits of agreement −0.87 to 1.1 beats/min, CC^2^ of 0.9973 and RMSE of 0.51 beats/min, while (Fig. 8) revealed a mean bias of 0.15 beats/min with 95% limits of agreement −1.5 to 1.8 beats/min, CC^2^ of 0.9926 and RMSE of 0.84 beats/min. Based on ICA and PCA, the statistics were 0.38; −2.5 to 3.3; 0.9759; 1.53 beats/min based ICA (see Fig. 8c) and 0.4; −3.1 to 3.9; 0.965; 1.83 beats/min based on PCA (see Fig. 8d) for the main bias, limits of agreement, CC^2^ and RMSE respectively. The Bland–Altman plots for the last scenario are shown in Fig. 9.

The Bland–Altman plot (Fig. 9a) showed the statistics were 0.17, −1.6 to 1.9, 0.9917 and 0.89 beats/min for the mean bias, limits of agreement, CC^2^ and RMSE respectively when the proposed system with magnification process was used, while Fig. 9b showed the statistics were 0.24, −2.1 to 2.6, 0.9848 and 1.2 beats/min respectively when the proposed system without magnification process was used instead. The statistics based on ICA were 0.58, −5.1 to 6.3, 0.9089 and 2.94 beats/min (see Fig. 9c), whereas they were 0.6, −5.7 to 6.9, 0.8887 and 3.24 beats/min based on PCA (see Fig. 9d).

**Fig. 10:**
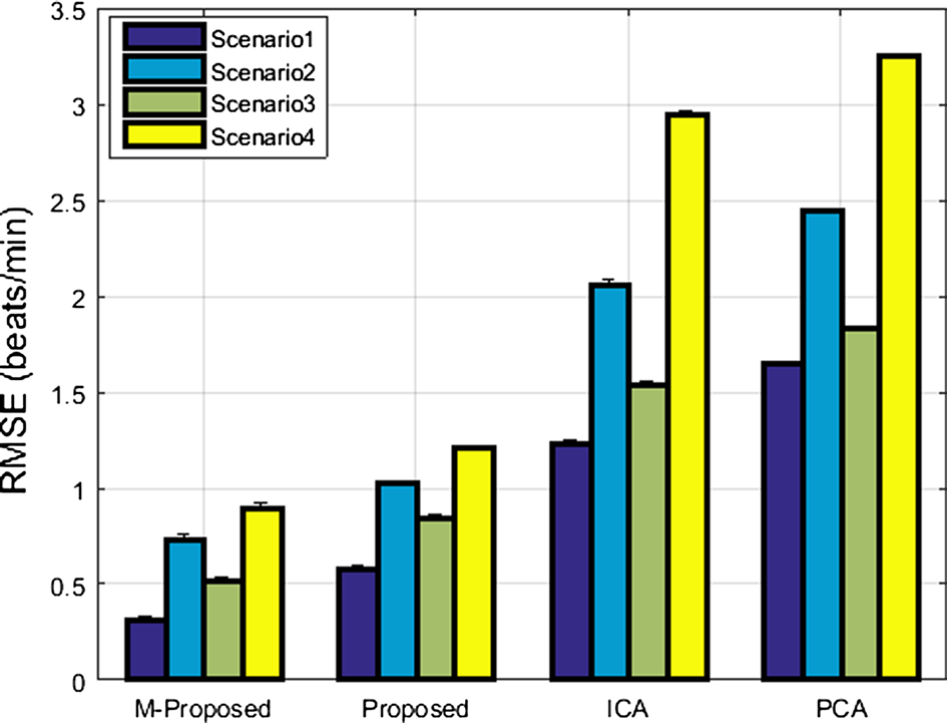
RMSE performance of various heart rate measuring systems for all proposed scenarios

A performance comparison of various measuring systems based on their RMSE value for the detection of heart rate for all proposed scenarios is shown in Fig. 10.

### Respiratory rate measurements

**Fig. 11:**
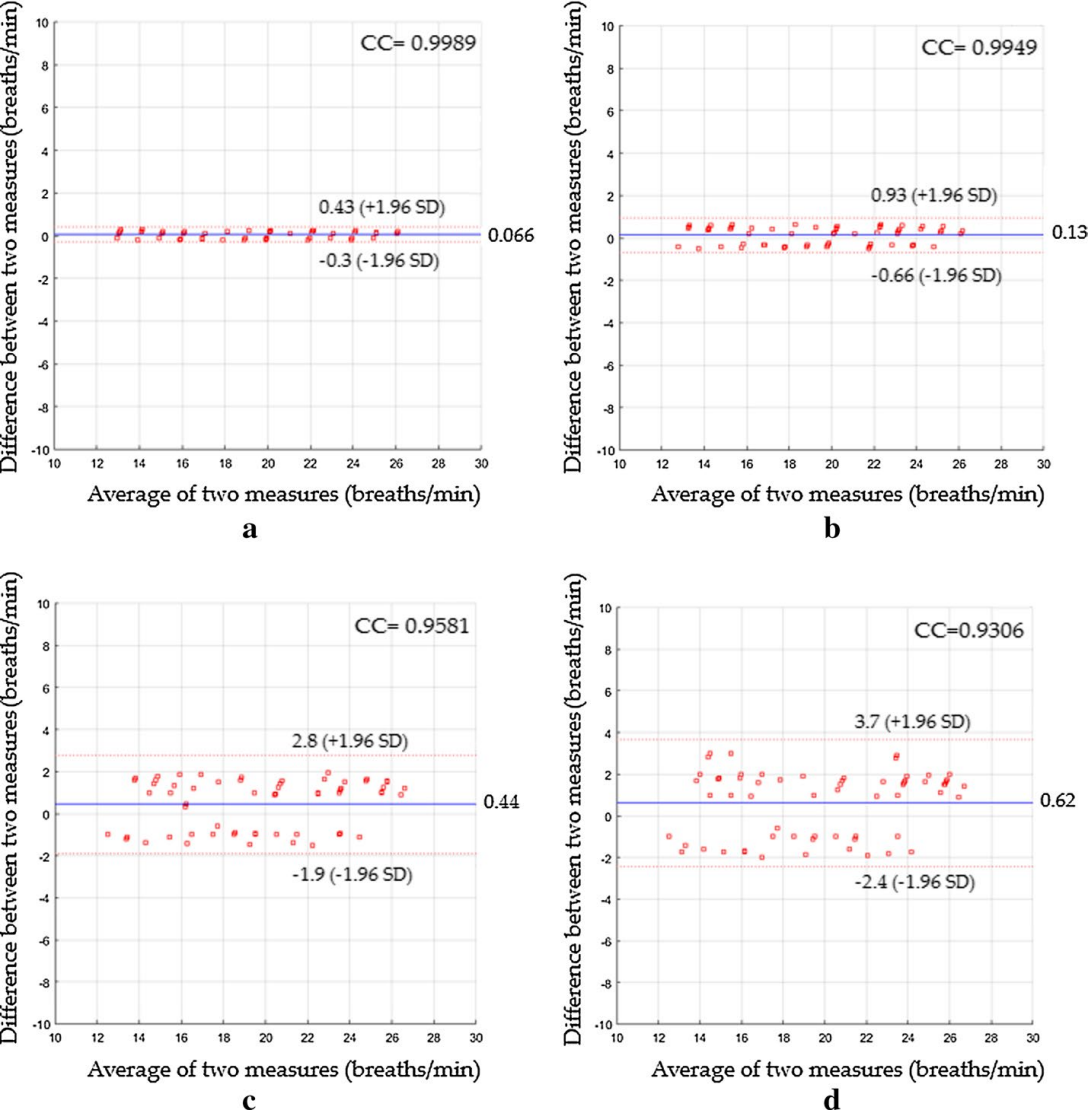
Bland–Altman plots between respiratory rate measurements obtained by reference method and respiratory rates measured by **a** the proposed system with magnification, **b** the proposed system without magnification, **c** ICA and **d** PCA for the first scenario

Figure 11 demonstrates a Bland–Altman plots of the respiratory rate measurements in the first scenario. The Bland–Altman plot (Fig. 11a) revealed a strong agreement between the difference between heart rate measurements by the proposed system using the magnification process and the reference measurements by the Piezo respiratory belt. The mean bias was 0.066 breaths/min and 95% limit of agreement range between −0.3 and 0.43 breaths/min with a CC^2^ of 0.9978 and a RMSE of 0.2 breaths/min. Bland–Altman plot (Fig. 11b) revealed a mean bias of 0.13 breaths/min, agreement range between −0.66 and 0.93 breaths/min, a CC^2^ of 0.9898 and a RMSE of 0.42 breaths/min when the proposed system without magnification process was used instead. Using ICA as shown in Fig. 11c, the main bias was 0.44 breaths/min with agreement range between −1.9 and 2.8 breaths/min. The CC^2^ was 0.918 and the RMSE was 1.26 breaths/min. Using PCA as shown in Fig. 11d, the main bias was 0.62 breaths/min with agreement range between −2.4 and 3.7 breaths/min. The CC^2^ was 0.8661 and the RMSE was 1.66 breaths/min.

**Fig. 12:**
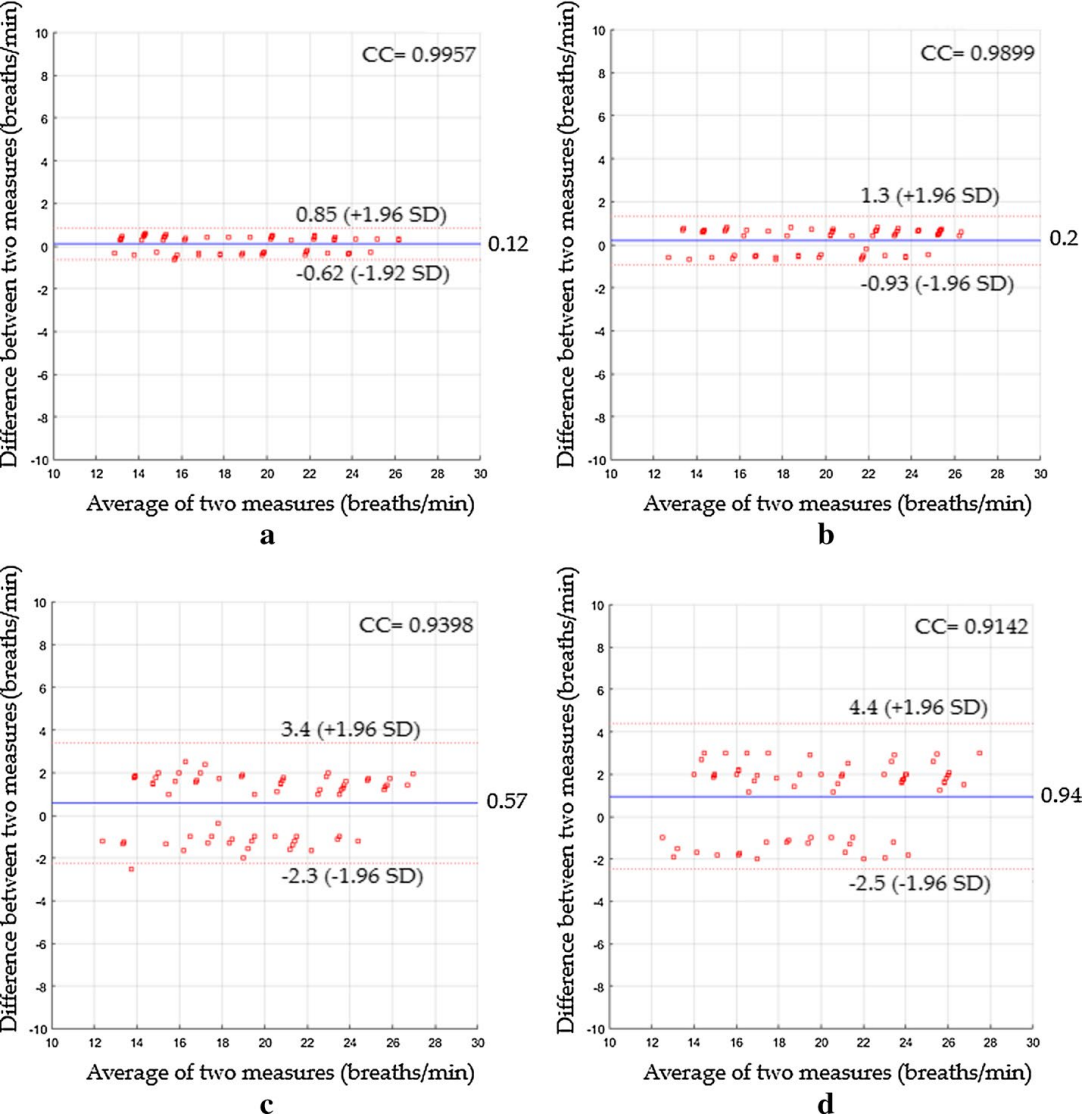
Bland–Altman plots between respiratory rate measurements obtained by reference method and respiratory rates measured by **a** the proposed system with magnification, **b** the proposed system without magnification, **c** ICA and **d** PCA for the second scenario

In the second scenario, Fig. 12a revealed a mean bias of 0.12 breaths/min with agreement range between −0.62 to 0.85 breaths/min, a CC^2^ of 0.9913 and a RMSE of 0.39 breaths/min, while Fig. 12b revealed a mean bias of 0.2 breaths/min with agreement range between −0.93 to 1.3 breaths/min, CC^2^ of 0.9799 and RMSE of 0.6 breaths/min. Using ICA as shown in Fig. 12c, the statistics were 0.57 breaths/min of a mean bias; −2.3 to 3.4 breaths/min agreement range; 0.8833 of CC^2^; 1.54 breaths/min of RMSE, whereas when PCA was used, the statistics were 0.94 breaths/min; −2.5 to 4.4 breaths/min agreement range; 0.8358 of CC^2^; 1.98 breaths/min of RMSE as shown in Fig. 12d.

**Fig. 13:**
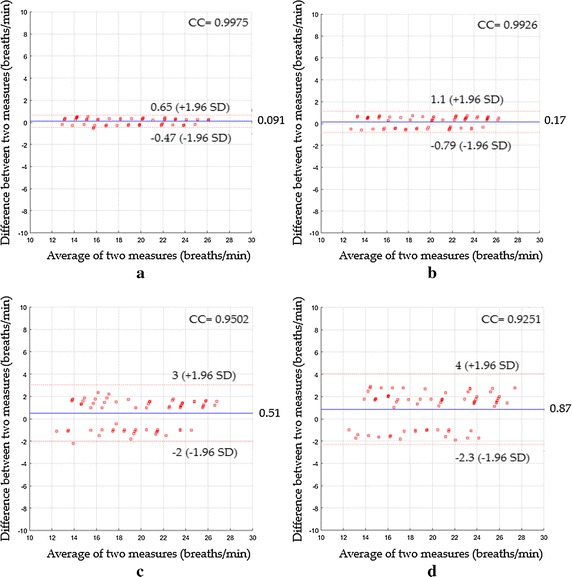
Bland–Altman plots between respiratory rate measurements obtained by reference method and respiratory rates measured by **a** the proposed system with magnification, **b** the proposed system without magnification, **c** ICA and **d** PCA for the third scenario

In the third scenario, Fig. 13a showed a mean bias of 0.091 breaths/min with agreement range between −0.47 to 0.65 breaths/min, a CC^2^ of 0.995 and a RMSE of 0.3 breaths/min, while Fig. 13b showed a mean bias of 0.17 breaths/min with agreement range between −0.79 to 1.1 breaths/min, CC^2^ of 0.9853 and RMSE of 0.52 breaths/min. Using ICA as shown in Fig. 13c, the statistics were 0.51 breaths/min of a mean bias; −2 to 3 breaths/min agreement range; 0.9028 of CC^2^; 1.38 breaths/min of RMSE, whereas when PCA was used, the statistics were 0.87; −2.3 to 4 breaths/min agreement range; 0.8558 of CC^2^; 1.83 breaths/min of RMSE as shown in Fig. 13d.

**Fig. 14:**
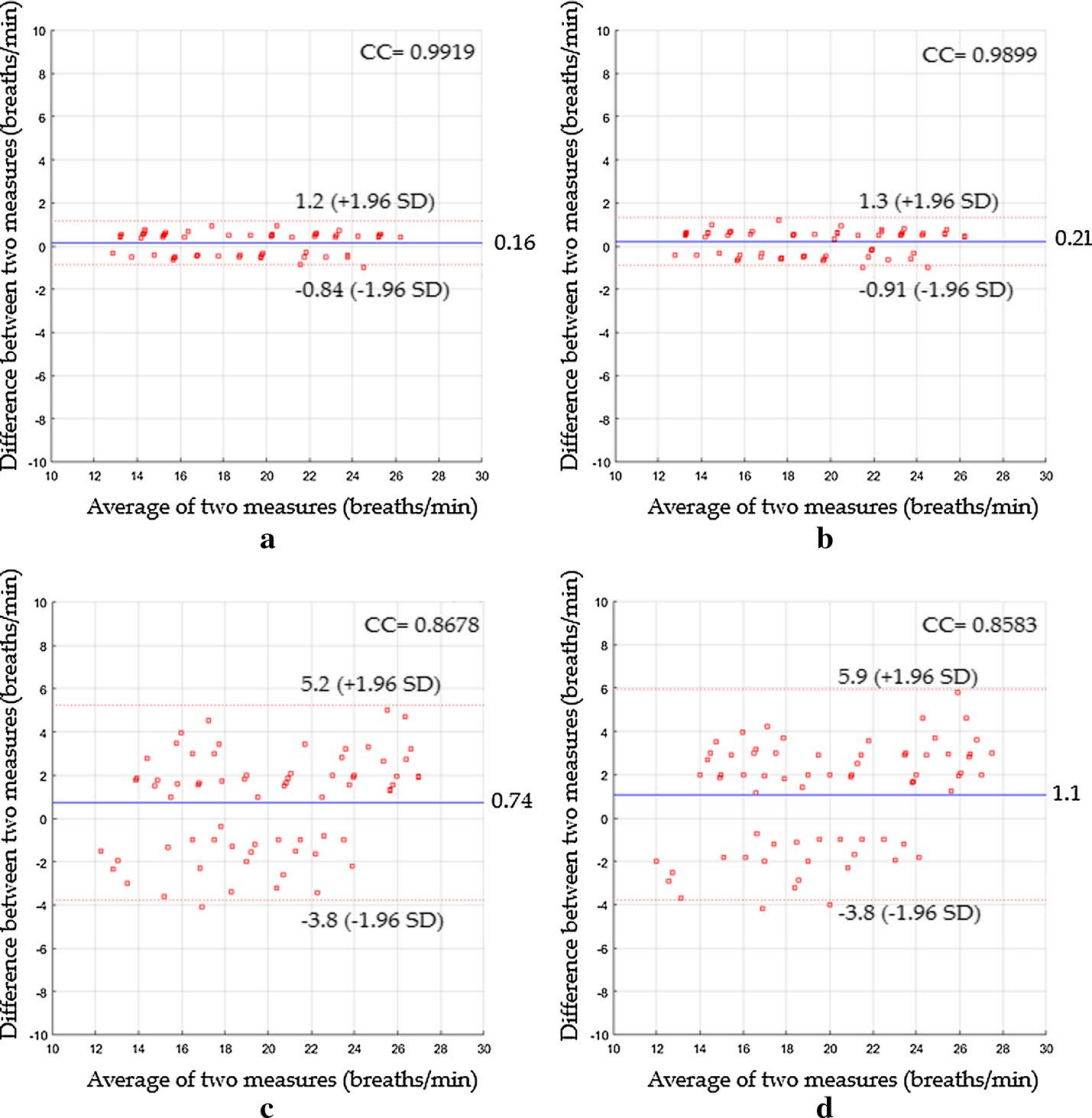
Bland–Altman plots between respiratory rate measurements obtained by reference method and respiratory rates measured by **a** the proposed system with magnification, **b** the proposed system without magnification, **c** ICA and **d** PCA for the fourth scenario

In the fourth scenario, Fig. 14a indicated a mean bias of 0.16 breaths/min with agreement range between −0.84 to 1.2 breaths/min, a CC^2^ of 0.9838 and a RMSE of 0.53 breaths/min, while Fig. 14b showed a mean bias of 0.21 breaths/min with agreement range between −0.91 to 1.3 breaths/min, CC^2^ of 0.98 and RMSE of 0.6 breaths/min. Using ICA as shown in Fig. 14c, the statistics were 0.74 breaths/min of a mean bias; −3.8 to 5.2 breaths/min agreement range; 0.7531of CC^2^; 2.4 breaths/min of RMSE, whereas when PCA was used, the statistics were 1.1; −3.8 to 5.9 breaths/min agreement range; 0.7366 of CC^2^; 2.69 breaths/min of RMSE as shown in Fig. 14d.

**Fig. 15:**
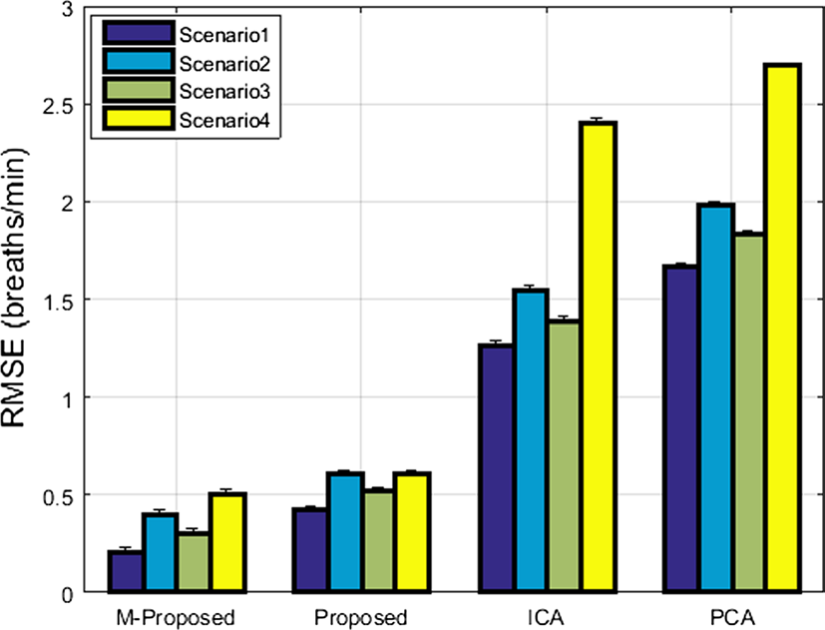
RMSE performance of various respiratory rate measuring systems for all proposed scenarios

A performance comparison of various measuring systems based on their RMSE value for the detection of respiratory rate for all proposed scenarios is shown in Fig. 15.

## Discussion

The results show that PPG can be successfully performed from a hovering UAV if a suitably selective spatiotemporal motion detection scheme is used. The experimental results on many video sequences show that the estimated heart and respiratory rates had high agreement with the reference methods and outperformed the state of the art methods (ICA and PCA) in four different proposed scenarios.

In the stationary scenario, the proposed system with the magnification process showed an excellent agreement with the reference method [CC^2^ = 0.9991, RMSE = 0.31 beats/min and mean error (ME) = 0.29%] with respect to the heart rate measurements and (CC^2^ = 0.9978, RMSE = 0.2 breaths/min and ME = 0.18%) with respect to the respiratory rate measurements. Our proposed system without the magnification process could also measure these vital signs with a very good agreement (CC^2^ = 0.9966, RMSE = 0.57 beats/min, and ME = 0.54% for heart rate measurements and CC^2^ = 0.9898, RMSE = 0.42 breaths/min, and ME = 0.41% for the respiratory rate measurements). It clear that our system with and without the magnification process reduced the mean bias, limit of agreement and RMSE as well as increased the correlation level compared to when ICA and PCA were used instead to extract vital signs from the full face area. ICA under the first scenario had a ME of 1.18% for heart rate and 1.22% for respiratory rate, whereas PCA had a ME of 1.54% for heart rate and 1.58% for respiratory rate.

In the second scenario (face expression and head rotation), the proposed system with the magnification process also had very good agreement with the reference method (CC^2^ = 0.9944, RMSE = 0.73 beats/min, and ME = 0.72%) with respect to the heart rate measurements and (CC^2^ = 0.9913, RMSE = 0.39 breaths/min, and ME = 0.38%) with respect to the respiratory rate measurements which were slightly better than when we used our system without the magnification process (CC^2^ = 0.989, RMSE = 1.02 beats/min, and ME = 0.99% for heart rate measurements and CC^2^ = 0.9799, RMSE = 0.6 breaths/min, and ME = 0.59% for the respiratory rate measurements). This is significantly better than the statistics achieved when ICA and PCA were used instead. ICA under the second scenario had a ME of 1.98% for heart rate and 1.49% for respiratory rate, whereas PCA had a ME of 2.37% for heart rate and 1.89% for respiratory rate.

In the third scenario (talking), our results with and without magnification process also had a better correlation than those obtained from the ICA and PCA. The statistics (CC^2^, RMSE, and ME) with the magnification process were 0.9973, 0.51 beats/min and 0.5% respectively for heart rate and 0.995, 0.3 breaths/min and 0.29% for respiratory rate, whereas without using the magnification process, they were 0.9926, 0.84 beats/min and 0.82% for the heart rate and 0.9853, 0.52 breaths/min and 0.5% for the respiratory rate. Under this scenario, ME based-ICA was 1.5% for the heart rate and 1.34% for the respiratory rate, whereas ME based-PCA was 1.79% for the heart rate and 1.75% for the respiratory rate.

Our results with and without the magnification process under the last scenario (lighting condition) also exhibited very good correlation and low RMSE compared to ICA and PCA which might fail in extracting the heart and respiratory rates with low correlation levels and high RMSE. The statistics (CC^2^, RMSE, and ME) based on the magnification process were 0.9917, 0.89 beats/min and 0.88% for heart rate, and 0.9838, 0.53 breaths/min and 0.52% for respiratory rate, whereas they were 0.9848, 1.2 beats/min and 1.18% for heart rate and 0.98, 0.6 breaths/min and 0.59% for respiratory rate without the magnification process. ICA under the fourth scenario had a ME of 2.78% for heart rate and 2.17% for respiratory rate, whereas PCA had a ME of 3.05% for heart rate and 2.49% for respiratory rate.

For the all proposed scenarios, our system with the magnification process presented a CC^2^ of 0.9956, RMSE of 0.65 beats/min, and ME of 0.6% for heart rate measurements and a CC^2^ of 0.9919, RMSE of 0.38 breaths/min, and ME of 0.34% for respiratory rate measurements, whereas the results obtained without magnification process produced a CC^2^ of 0.9907, RMSE of 0.94 beats/min and ME of 0.88% for heart rate measurements and a CC^2^ of 0.9837, RMSE of 0.5 breaths/min, and ME of 0.52% for respiratory rate measurements. Using ICA, the statistics (CC^2^, RMSE, and ME) were 0.956, 2.04 beats/min and 1.86% respectively for heart rate measurements, and 0.8188, 1.97 breaths/min, and 1.77% respectively for respiratory rate measurements, whereas when PCA was used instead, they were 0.9405, 2.37 beats/min and 2.19% respectively for heart rate measurements, and 0.8164, 2.07 breaths/min, and 1.93% respectively for respiratory rate measurements.

We also tested the computational time of the proposed noise artifact removal method based on CEEMDAN-CCA against the ICA and PCA. The mean computational time for CEEMDAN-CCA with 200 iterations for 30 s iPPG signal was 1.22 s, while the means for ICA and PCA were 0.86 and 0.79 s respectively. The implementation was carried out in the MATLAB program (2015b) and run under Microsoft Windows 10 (64 bits) on a computer with Intel Quad Core i5-4570 3.20 GHz CPU and 8.00 GB of RAM. The computational time cost is acceptable for noise artifact removal from the iPPG signal, which makes it suitable for real-time applications. It also noted that our proposed system does not require extra hardware to stream the video since the UAV contains some software modules to facilitate communications through Wi-Fi and provides logging capability which makes real-time processing more flexible and feasible.

The potential estimation of other important vital signs such as heart rate variability and blood oxygen saturation level (SpO_2_) is an important future work. The SpO_2_ can be extracted from the iPPG signal captured by a digital camera at two different wavelengths based on ac/dc component analysis instead of direct image intensity analysis of the iPPG signal used in this study.

## Conclusion

For the first time, we have shown that video from a hovering UAV can be used to measure cardiorespiratory signals. We have used a combination of both CEEMDAN and CCA techniques to remove noise acquired from the illumination variations, subject’s movement and camera movement. Also, we have demonstrated that the heart and respiratory rates can efficiently be extracted based on the proposed system with and without the developed video magnification system. The experimental results obtained from 15 subjects in different scenarios showed that the estimated heart and respiratory rates were very close to the reference methods (finger pulse oximeter and Piezo respiratory belt transducer) with very low RMES and ME. Furthermore, the proposed system significantly outperformed the state-of-the-art methods such as ICA and PCA. Therefore, the proposed system is a feasible solution to remove the noise effects resulting from the illumination variations, subject’s movement and camera movement from the iPPG signals and may be a promising approach in realistic non-contact vital signs measurement applications. Future work will consider techniques that may be more robust in the presence of UAV and target locomotion and changes in pose.

## Abbreviations

iPPG: imaging photoplethysmography
UVA: unmanned aerial vehicle
CEEMDAN: complete ensemble empirical mode decomposition with adaptive noise
CCA: canonical correlation analysis
ICA: independent component analysis
PCA: principle component analysis
FFT: fast Fourier transform
BSS: blind source separation
SD: standard deviation
CC^2^: squared correlation coefficients
RMSE: root mean squared error
ME: mean error rate
